# A Systematic Review and Meta-Analysis of Inpatient Mortality Associated With Nosocomial and Community COVID-19 Exposes the Vulnerability of Immunosuppressed Adults

**DOI:** 10.3389/fimmu.2021.744696

**Published:** 2021-10-06

**Authors:** Mark J. Ponsford, Tom J. C. Ward, Simon M. Stoneham, Clare M. Dallimore, Davina Sham, Khalid Osman, Simon M. Barry, Stephen Jolles, Ian R. Humphreys, Daniel Farewell

**Affiliations:** ^1^ Immunodeficiency Centre for Wales, University Hospital for Wales, Cardiff, United Kingdom; ^2^ Henry Wellcome Building, Division of Infection & Immunity, School of Medicine, Cardiff University, Cardiff, United Kingdom; ^3^ Department Respiratory Sciences, College of Life Sciences, University of Leicester, Leicester, United Kingdom; ^4^ Institute for Lung Health, National Institute for Health Research (NIHR) Leicester Biomedical Research Centre, Glenfield Hospital, Leicester, United Kingdom; ^5^ Department of Global Health and Infection, Brighton and Sussex Medical School, University of Sussex, Brighton, United Kingdom; ^6^ Department of Microbiology and Infection, Royal Sussex County Hospital, Brighton, United Kingdom; ^7^ Department of Anaesthetics, University Hospital for Wales, Cardiff, United Kingdom; ^8^ Department of Neonatology, University Hospitals of Leicester National Health Service (NHS) Trust, Leicestershire, United Kingdom; ^9^ Department of Respiratory Medicine, Cardiff and Vale University Health Board, Cardiff, United Kingdom; ^10^ Respiratory Health Implementation Group, Swansea University, Swansea, United Kingdom; ^11^ Systems Immunity Research Institute, School of Medicine, Cardiff University, Cardiff, United Kingdom; ^12^ Division of Population Medicine, School of Medicine, Cardiff University, Cardiff, United Kingdom

**Keywords:** covid-19, nosocomial transmission, immunodeficiency, hospital-acquired, infection control

## Abstract

**Background:**

Little is known about the mortality of hospital-acquired (nosocomial) COVID-19 infection globally. We investigated the risk of mortality and critical care admission in hospitalised adults with nosocomial COVID-19, relative to adults requiring hospitalisation due to community-acquired infection.

**Methods:**

We systematically reviewed the peer-reviewed and pre-print literature from 1/1/2020 to 9/2/2021 without language restriction for studies reporting outcomes of nosocomial and community-acquired COVID-19. We performed a random effects meta-analysis (MA) to estimate the 1) relative risk of death and 2) critical care admission, stratifying studies by patient cohort characteristics and nosocomial case definition.

**Results:**

21 studies were included in the primary MA, describing 8,251 admissions across 8 countries during the first wave, comprising 1513 probable or definite nosocomial COVID-19, and 6738 community-acquired cases. Across all studies, the risk of mortality was 1.3 times greater in patients with nosocomial infection, compared to community-acquired (95% CI: 1.005 to 1.683). Rates of critical care admission were similar between groups (Relative Risk, RR=0.74, 95% CI: 0.50 to 1.08). Immunosuppressed patients diagnosed with nosocomial COVID-19 were twice as likely to die in hospital as those admitted with community-acquired infection (RR=2.14, 95% CI: 1.76 to 2.61).

**Conclusions:**

Adults who acquire SARS-CoV-2 whilst already hospitalised are at greater risk of mortality compared to patients admitted following community-acquired infection; this finding is largely driven by a substantially increased risk of death in individuals with malignancy or who had undergone transplantation. These findings inform public health and infection control policy and argue for individualised clinical interventions to combat the threat of nosocomial COVID-19, particularly for immunosuppressed groups.

**Systematic Review Registration:**

PROSPERO CRD42021249023

## 1 Introduction

Health-care-associated infections represent an enduring and serious threat to patient safety (1, 2), and are estimated to cost the National Health Service (NHS) £1 billion each year (3). The transmission of respiratory viruses such as influenza in the healthcare environment are a well-recognized cause of significant morbidity and mortality at the individual patient level (4), however less is known regarding the significance of in-hospital (nosocomial) transmission of the novel pandemic coronavirus SARS-CoV-2 causing COVID-19 (5). Since its emergence in 2019, COVID-19 has placed enormous pressure on health-care systems worldwide. Limited availability of testing, asymptomatic infections, and an evolving understanding of routes of transmission have led to the exposure of potentially vulnerable uninfected patients in the health-care setting (6).

The first and only rapid literature review and meta-analysis conducted to date on nosocomial COVID-19 in hospitalised individuals was published in April 2020, early in the course of the pandemic, and included only 3 studies reporting prevalence (7). The UK COVID-19 Clinical Information Network (CO-CIN) estimated 31,070 nosocomial COVID-19 infections occurred in England between February and July 2020, but made no assessment of the risk of mortality (8). We recently reported our initial experience from the first wave of the COVID-19 pandemic across the nation of Wales, using data collected from 2508 hospitalised adults (9). In this observational study, inpatient mortality rates for nosocomial COVID-19 ranged from 38% to 42% and were consistently higher than participants with community-acquired infection (31% to 35%) across a range of possible case definitions. Whilst supported by other studies (10, 11), this finding contrasts with several earlier reports suggesting that nosocomial COVID-19 infection is associated with a similar risk of inpatient mortality to community acquired infection (12–14).

It is well known that individuals with pre-existing health conditions particularly ischemic heart disease, diabetes, hypertension and immunosuppression (15–17), as well as older and frailer individuals (18), are at increased risk of death from SARS-CoV-2. Such individuals are also likely to be over-represented in inpatient cohorts (19). Together, this suggests a robust assessment of the burden of mortality is urgently needed to examine the risk to patients, identify vulnerable cohorts, and direct policies to ensure improvement. We therefore performed a systematic review and meta-analysis of published and pre-print studies reporting mortality associated with probable and definite nosocomial SARS-CoV-2 outbreaks during the first wave of the COVID-19 pandemic. Our primary aim was to describe and compare case fatality rates associated with nosocomial- and community-acquired COVID-19 cases within hospitalised adults. Our secondary aims were to assess the variation in risk of mortality between patient sub-groups, the relative risk of critical care admissions, and to probe the risk of bias associated with these reports. Together, this provides a timely insight to the global burden of hospital-acquired COVID-19 and highlight key patient groups at elevated risk of mortality. Thus, although we do not provide a direct assessment of the causal contribution of nosocomial exposure to the risk of death, these findings inform public health policy and argue for enhanced infection control alongside access to post-exposure interventions for those at high risk of severe COVID-19 during their healthcare interactions.

## 2 Methods

We followed the Preferred Reporting Items for Systematic Reviews and Meta-Analyses (PRISMA) 2020 (20). The study protocol was prospectively registered with Prospero (CRD42021249023), having first confirmed no similar reviews were underway.

### 2.1 Eligibility Criteria

#### 2.1.1 Participants

Studies of hospitalised adults (≥16 years) within acute or long-term healthcare settings, excluding care or residential homes. We specifically focused on outcomes for hospitalised adults and excluded outcomes from health care workers with nosocomial infection, as the latter has been recently evaluated (21).

#### 2.1.2 Exposures

We included any implicit or explicit case definition of probable or definite nosocomial acquisition as defined by the study authors, considering these further in sensitivity analyses. Patients where COVID-19 origin was unclassified were excluded. Implementation of universal screening of patients and healthcare workers, and changes to personal protective equipment have recently been reported in detail elsewhere (22) and were not further considered.

#### 2.1.3 Comparators

The number and outcome of adults hospitalised with community-acquired SARS-CoV-2 within the same study setting.

#### 2.1.4 Outcomes

The primary outcome was mortality of nosocomial SARS-CoV-2 infections in hospitalised adult patients and community-acquired SARS-CoV-2 infection. Secondary outcomes included rates of critical care admission, and qualitative analysis of case definitions, study timing, and variation in reporting by country of origin.

#### 2.1.5 Study Design

Observational case series and cohort studies were included, provided they reported an outbreak of nosocomial SARS-CoV-2 (defined as ≥2 patients with likely nosocomial infection) within the hospital setting. Case reports with a single participant (high risk of bias, unable to assess proportion/risk), exclusively outpatient populations (e.g., dental practice), and non-patient populations (e.g., healthcare workers only) were therefore excluded.

### 2.2 Search Strategy to Identify Studies

#### 2.2.1 Database Search Strings

Ovid Medline, Embase, and the Social Policy & Practice databases and MedRvix.org were searched from 1/1/2020 to 9/2/2021. A search string was designed that included the following concepts: [SARS-CoV-2 OR sars-cov 2 OR COVID-19 OR covid 19 OR 2019-nCoV or “COVID-19”] AND [nosocomial OR hospital-acquire* or nosocomial-acquire* OR cross infection].

#### 2.2.2 Restriction on Publication Type

No restrictions by language were imposed, and Google Translate was used to review full text documents where required. In addition to considering full-text articles, publications available as abstract only were included if they contained sufficient information to inform the primary outcome.

#### 2.2.3 Study Selection and Screening

**Figure 1:**
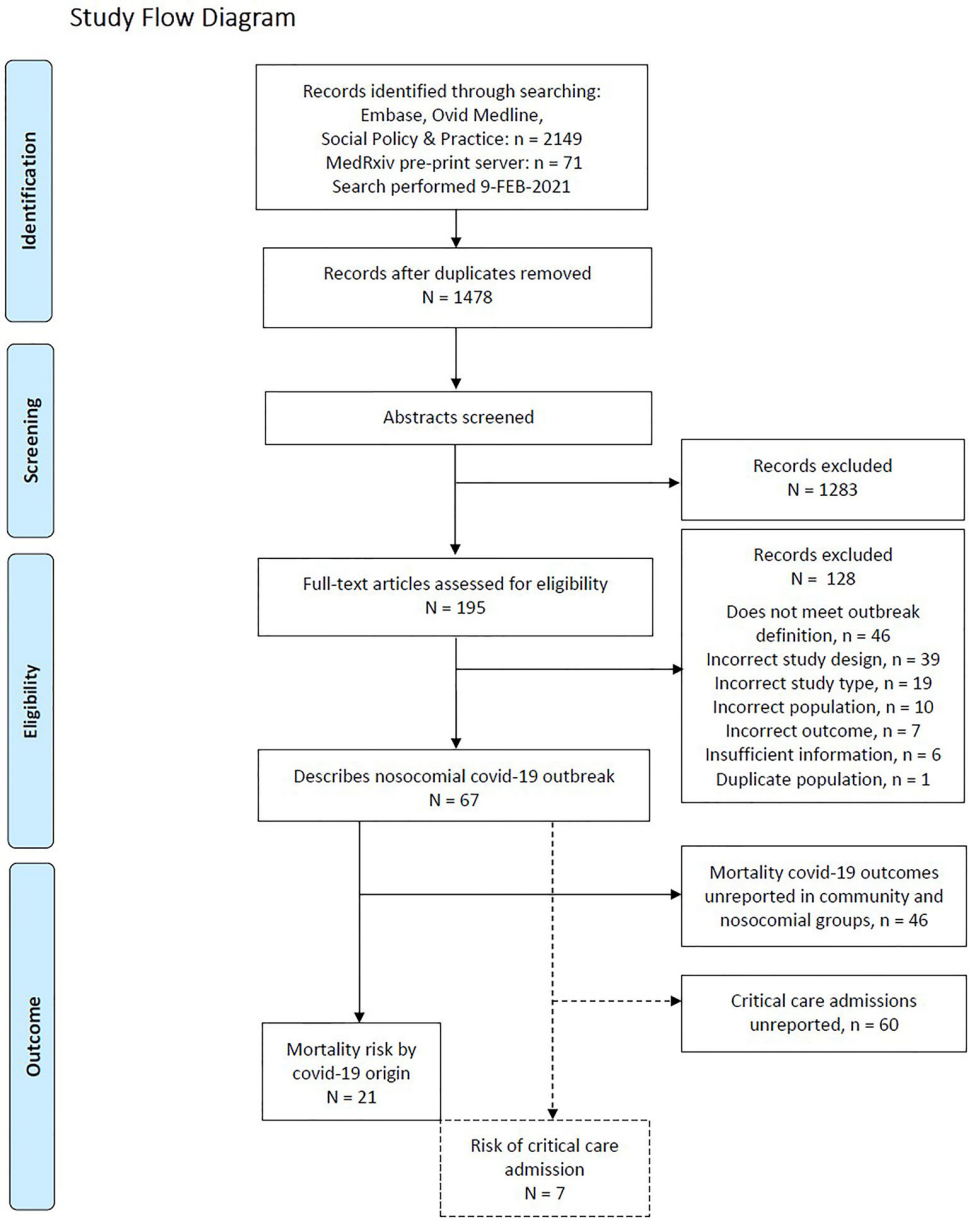
PRISMA Study Flow Diagram.

Five clinicians (MJP, TJCW, SS, DS, KO, CD) independently screened titles and abstracts against inclusion criteria using Rayyan (23). MJP retrieved the full-texts, and with TJCW and SS screened these for inclusion. Conflicts were resolved by consensus. The selection process is outlined in the PRISMA flow diagram (**Figure 1**).

### 2.3 Data Extraction

Data was extracted using a pre-defined spreadsheet with fields as presented in **Table 1** and cross-checked for accuracy and completeness by a second reviewer. COVID-19 case diagnosis rates by country were retrieved from https://ourworldindata.org/coronavirus-source-data on 6th April 2021. Pre-print articles subsequently accepted by peer-reviewed journals were used for analysis.

**Table 1:** Evidence summary table.

Reference	Study type	Country	Study population and setting	Study period ^x^	Nosocomial case definition	Number of participants (%)^†^, *	Mortality (%)^†^	Critical care admission	Length of follow-up
Ajayi et al. (24)	Retrospective cohort	UK	39 hospitalized adult trauma patients with RT-PCR diagnosis of COVID-19 admitted to London centre.	26/1/20 to 14/4/20 (80 days)	No explicit definition.	Community: 12 (30.8%) Nosocomial: 27 (69.2%)	Community: 1 (8.3%) Nosocomial: 7 (25.9%)	Not reported.	Until death or discharge.
Bhogal et al. (25)	Retrospective cohort	UK	179 hospitalized adult cancer patients with RT-PCR diagnosis of COVID-19 across 6 hospitals in England.	1/3/20 to 10/6/20 (102 days)	“Probable”: 8-14 days. “Definite”: > 14 days following admission	Community: 145 (82.8%) Nosocomial: 28 (16.2%)	Community: 36 (24.8%) Nosocomial: 18 (64.3%)	Not reported.	Until discharge, death, or last available follow-up 17/6/20 (minimum 7 days; median 44).
Brill et al. (26)	Retrospective cohort	UK	450 hospitalized adults with RT-PCR diagnosis of COVID-19 in London teaching hospital	10/3/20 to 8/4/20 (30 days)	RT-PCR diagnosis made >14 days following continuous admission.	Community: 419 (93.1%) Nosocomial: 31 (6.9%)	Community: 166 (39.6%) Nosocomial: 7 (22.6%)	Not reported.	Until death or discharge.
Cao et al. (27)	Retrospective cohort	China	78 adults hospitalized with laboratory-confirmed COVID-19 in Wuhan (24 healthcare workers excluded)	3/1/20 to 1/2/20 (30 days)	Close contact with known positive case whilst admitted to hospital or outpatient visit in last 14 days	Community: 68 (87.2%) Nosocomial: 10 (12.8%)	Community: 15 (22.1%) Nosocomial: 2 (20.0%)	Not reported.	Until death or discharge, until 15/2/20 (minimum 14 days).
Carter et al. (12)	Prospective cohort	UK and Italy	1564 hospitalized adults with laboratory-confirmed COVID-19 across 10 UK and 1 Italian hospitals	27/2/20 to 28/4/20 (62 days)	“Definite”: > 14 days from admission to diagnosis.	Community: 1368 (87.5%) Nosocomial: 196 (12.5%)	Community: 372 (27.2%) Nosocomial: 53 (27.0%)	Not reported.	Until death or discharge (minimum 7 days).
Coll et al. (28)	Retrospective case series	Spain	778 solid organ transplant and hematopoietic stem cell transplant recipients with clinical-laboratory COVID-19 diagnosis across 61 Spanish transplant centres.	20/2/20 to 13/7/20 (145 days)	No explicit definition given.	Community: 679 (87.3%) Nosocomial: 99 (12.7%)	Community*: 133 of 570 (23.3%) Nosocomial*: 37 of 77 (48.1%)	Not reported.	Not explicitly defined. *Outcome data available in 647 only.
Davis et al. (29)	Retrospective cohort	UK	222 hospitalized adults with a RT-PCR confirmed diagnosis of COVID-19 within department of medicine for elderly across 3 Scottish (UK) hospitals	18/3/20 to 20/4/20 (34 days)	RT-PCR diagnosis made >14 days following admission.	Community: 119 (53.6%) Nosocomial: 103 (46.4%)	Community: 54 (45.4%) Nosocomial: 41 (39.8%)	Community: 0 (0.0%) Nosocomial: 4 (3.9%)	30-day mortality following date of RT-PCR testing
Elkrief et al. (11)	Prospective cohort	Canada	249 hospitalized adults with cancer and a laboratory-confirmed diagnosis of COVID-19 (3 children excluded)	3/3/20 to 23/5/20 (82 days)	Diagnosis of COVID-19 >6 days after unrelated admission.	Community: 202 (81.1%) Nosocomial: 47 (18.9%)	Community: 49 (24.3%) Nosocomial: 22 (46.8%)	Community: 27 (13.4%) Nosocomial: 6 (12.8%)	Until death or last follow-up (median 25 days).
Garatti et al. (30)	Retrospective case series	Italy	10 hospitalized adults undergoing urgent cardiac surgery in Italian with a clinical diagnosis of COVID-19	21/2/20 to 08/03/20 (17 days)	Clinical diagnosis made > 8 days following admission.	Community: 4 (40%) Nosocomial: 6 (60%)	Community: 1 (25.0%) Nosocomial: 0 (0.0%)	Community: 1 (25.0%) Nosocomial: 0 (0.0%)	Until death or discharge (median 25 days post symptom onset).
Gonfiotti et al. (31)	Retrospective case series	Italy	5 adult patients hospitalized in Italian thoracic surgery unit with a RT-PCR confirmed diagnosis of COVID-19.	29/1/20 to 4/3/20 (36 days)	Close contact with known positive case whilst in hospital (no explicit interval defined).	Community: 1 (20.0%) Nosocomial: 4 (80.0%)	Community: 0 (0.0%) Nosocomial: 2 (50.0%)	Community: 0 (0.0%) Nosocomial: 1 (25.0%)	Until death or discharge (21-60 days post surgery).
Harada et al. (32)	Prospective cohort	Japan	562 patients tested prior or during hospitalization to Japanese university hospital following nosocomial outbreak.	24/3/20 to 24/4/20 (32 days)	Development of symptoms and RT-PCR test >5 days following admission.	Community: 19 (79.2%) Nosocomial: 5 (20.8%)	Community: 1 (5.3%) Nosocomial: 3 (60.0%)	Community: 4 (21.1%) Nosocomial: 1 (20.0%)	Not explicitly defined
Jewkes et al. (33)	Retrospective case series	UK	133 adults admitted to an acute stroke unit within the UK with nosocomial COVID-19 outbreak.	12/3/20 to 5/5/20 (54 days)	Development of symptoms and RT-PCR test >14 days following admission.	Community: 13 (61.9%) Nosocomial: 8 (38.1%)	Community: 7 (53.8%) Nosocomial: 3 (37.5%)	Not reported.	Not explicitly defined
Khan et al. (13)	Prospective cohort	UK	173 adults hospitalized within 3 acute Scottish (UK) hospitals with an RT-PCR confirmed COVID-19 on 9/4/20.	9/4/20 to 9/5/20 (30 days)	RT-PCR diagnosis made >7 days following admission.	Community: 154 (89.0%) Nosocomial: 19 (11.0%)	Community: 28 (18.2%) Nosocomial: 4 (21.1%)	Community: 46 (29.9%) Nosocomial: 2 (10.5%)	30-day outcomes from admission or diagnosis, censored at discharge.
Khonyongwa et al. (34)	Retrospective cohort (prevalence)	UK	856 adults hospitalized for at least an overnight stay with RT-PCR confirmed COVID-19 within a London (UK) hospital, and no recent admission.	1/3/20 to 18/4/20 (48 days)	Development of symptoms and RT-PCR test >14 days following admission for non-COVID-19 indication.	Community: 716 (92.5%) Nosocomial: 58 (7.5%)	Community: 187 (26.1%) Nosocomial: 15 (25.9%)	Community: 232 (32.4%) Nosocomial: 13 (22.4%)	30-day outcomes
Lakhani et al. (35)	Retrospective case series (prevalence)	Spain	288 hospitalized adult trauma patients admitted to Spanish (UK) centre.	9/3/20 to 4/5/20 (57 days)	Development of symptoms and RT-PCR test >4 days following admission and <14 days of discharge for non-COVID-19 indication.	Community: 10 (34.5%) Nosocomial: 19 (65.5%)	Community: 5 (50.0%) Nosocomial: 7 (36.8%)	Not reported.	Minimum 14-days after discharge
Lee et al. (10)	Retrospective cohort.	Spain	98 adults aged ≥ 65 years hospitalized with RT-PCR confirmed COVID-19 to 4 Korean hospitals.	18/2/20 to 4/3/20 (16 days)	Diagnosis of COVID-19 during admission for unrelated illness.	Community: 86 (87.8%) Nosocomial: 12 (12.2%)	Community: 13 (15.1%) Nosocomial: 7 (58.3%)	Community: 14 (16.3%) Nosocomial: 2 (16.7%)	Death or discharge (minimum 14-days following admission)
Pellaud et al. (36),^±^	Retrospective cohort	Switzerland	196 patients hospitalized with laboratory confirmed COVID-19 across 5 hospitals within Fribourg region.	1/3/20 to 12/4/20 (43 days)	No explicit definition reported.	Community: 183 (93.4%) Nosocomial: 13 (6.6%)	Not reported	Community: 49 (26.8%) Nosocomial: 0 (0%)	30 days after onset of symptoms
Ponsford et al. (9)	Retrospective cohort	UK	2508 hospitalized adults with RT-PCR diagnosis of COVID-19 across 18 hospitals in Wales (UK)	1/3/20 to 1/6/20 (123 days)	“Probable”: > 7 days “Definite”: > 14 days from admission to diagnosis (multiple considered)	Community: 1784 (71.1%) Nosocomial: 724 (28.9%)	Community: 585 (32.8%) Nosocomial: 300 (41.4%)	Not reported.	Until death or discharge, until 20/11/20 (minimum follow-up 142 days).
Sanchez et al. (37)	Prospective cohort (prevalence)	Spain	143 adults admitted for urological surgery within 2 Spanish hospitals.	9/3/20 to 3/5/20 (56 days)	Development of symptoms ≥3 days of surgery and within 14 days of discharge.	Community: 2 (40.0%) Nosocomial: 3 (60.0%)	Community: 1 (50.0%) Nosocomial: 0 (0.0%)	Community: 1 (50.0%) Nosocomial: 0 (0.0%)	14-days following hospital discharge.
Snell et al. (38)	Prospective cohort	UK	574 consecutive adults hospitalized with RT-PCR confirmed COVID-19 to single London (UK) hospital.	13/3/20 to 31/3/20 (19 days)	“Probable”: > 7 days “Definite”: > 14 days from admission to diagnosis; additional viral genomic and epidemiological analysis.	Community: 471 (84.6%) Nosocomial: 86 (15.4%)	Community: 81 (16.9%) Nosocomial: 29 (33.7%)	Not reported.	Death or discharge (duration unclear).
Vanhems et al. (39)	Retrospective case series	France	7 adults hospitalized with RT-PCR confirmed COVID-19 to 24-bed geriatric ward within Lyon region.	29/2/20 to 14/3/20 (15 days)	No explicit definition reported.	Community: 2 (28.6%) Nosocomial: 5 (71.4%)	Community: 1 (50.0%) Nosocomial: 1 (20.0%)	Community: 0 (0.0%) Nosocomial: 0 (0.0%)	Death or discharge (including transfer to other hospitals)
Wake et al. (40)	Prospective cohort (prevalence)	UK	662 adults hospitalized with RT-PCR confirmed COVID-19 to London hospital trust.	11/3/20 to 12/5/20 (63 days)	“Probable”: > 7 days “Definite”: > 14 days from admission to diagnosis	Community: 573 (92.7%) Nosocomial: 45 (7.3%)	Community: 208 (36.3%) Nosocomial: 14 (31.1%)	Community: Not reported Nosocomial: 2 (4.4%)	Unclear (median length of stay stated as 33 days, IQR 22-55).

^x^Assumed to include end date unless otherwise specified by authors.

^†^In event of multiple case definitions for nosocomial infection, “probable” and “definite” case are both included.

*Healthcare workers and children were excluded wherever reported separately to patients (age ≥ 16 years).

^±^Data only included within secondary meta-analysis.

### 2.4 Assessment of Risk of Bias

Formal risk of bias on a study and outcome level were conducted using the Newcastle Ottawa Score (NOS) for cohort studies and Joanna Briggs Institute (JBI) tools for case series and prevalence studies (41), as recommended by the National Institute for Clinical Excellence (NICE) (42). Assessment was performed by 2 independent reviewers, with arbitration with a third as required. We defined adequate follow-up as ≥28 days, or complete follow-up until death or discharge, to account for the potential unequal time points in disease course at study entry between community and nosocomial patients. We considered principal areas likely to introduce bias, indicated by * in **Tables 2**–**4**, equating to a minimum score of 5 across tools. Briefly, these assessed quality of selection: a) representativeness of the average nosocomial or community-acquired covid-19 case within the patient group, b) ascertainment bias, c) sufficient description of study subjects and case definition – requiring an explicit nosocomial case definition given and applied; and quality of outcome assessment: a) sufficient follow-up, and b) adequacy of follow-up – requiring sufficient participants to have reached the pre-specified outcome at time of reporting.

**Table 2:** Risk of bias assessment - cohort studies (n = 8).

Study Author	Domain 1*	Domain 2*	Domain 3*	Domain 4	Domain 5	Domain 6	Domain 7*	Domain 8*	Total Score
Ajayi et al.	1	1	1	0	0	1	0	0	**4**
Brill et al.	1	1	1	0	0	1	1	0	**5**
Lee et al.	1	1	1	0	0	1	0	1	**5**
Bhogal et al.	1	1	1	0	1	1	1	0	**6**
Elkrief et al.	1	1	1	0	1	1	1	0	**6**
Carter et al.	1	1	1	0	1	1	1	1	**7**
Khan et al.	1	1	1	0	1	1	1	1	**7**
Ponsford et al.	1	1	1	0	1	1	1	1	**7**

^*^Indicate core quality domains, as considered in sensitivity analysis.

**Table 3:** Risk of bias assessment - prevalence studies (n = 8).

Study Author	Domain 1*	Domain 2*	Domain 3*	Domain 4*	Domain 5	Domain 6	Domain 7*	Domain 8	Total Score
Jewkes et al.	1	1	1	1	0	0	0	0	**4**
Wake et al.	1	0	1	1	0	0	1	0	**4**
Sanchez et al.	1	1	1	1	0	0	1	0	**5**
Harada et al.	1	1	1	1	1	1	0	0	**6**
Davis et al.	1	1	1	1	1	1	1	0	**7**
Cao et al.	1	1	1	1	1	1	1	0	**7**
Khonyongwa et al.	1	1	1	1	1	1	1	1	**8**
Lakhani et al.	1	1	1	1	1	1	1	1	**8**

^*^Indicate core quality domains, as considered in sensitivity analysis.

**Table 4:** Risk of bias assessment - case series (n = 5).

Study	Domain 1*	Domain 2*	Domain 3	Domain 4*	Domain 5	Domain 6	Domain 7	Domain 8*	Domain 9*	Domain 10	Total Score
Vanhems et al.	1	0	0	0	0	1	1	1	0	0	**4**
Snell et al.	1	1	1	1	0	0	0	1	1	0	**6**
Coll et al.	1	0	1	0	0	1	1	0	1	1	**6**
Gonfiotti et al.	0	1	1	1	0	1	1	1	1	0	**7**
Garatti et al.	1	0	0	1	1	1	1	1	1	1	**8**

^*^Indicate core quality domains, as considered in sensitivity analysis.

### 2.5 Data Analysis

Analysis was performed using R version 4.0.2 in RStudio (Version 1.3.959, R Foundation, Vienna, Austria) using the metafor package. Full details can be found within the online **Supplementary Methods**. Briefly, a random effects model was used to compare relative risk of mortality and ICU admission between patients with community-acquired and nosocomial COVID-19. Full details of the statistical methods used are available at https://cran.r-project.org/web/packages/metafor/metafor.pdf. Residual maximum likelihood (REML) was used to estimate the heterogeneity variance (τ^2^) (43). We conducted subgroup analyses based on classifications agreed by the reviewers reflecting the cohort best represented by the studies, i.e. in cohorts that were clinically and methodologically similar (44). Cochrane’s Q-test and I^2^ were used to assess the degree of inconsistency across studies (45, 46). Two-sided statistical significance was set at p<0.05. We conducted the following pre-specified sensitivity analyses:

- 1: Studies providing an explicit definition of nosocomial acquisition
- 2: Studies providing outcomes associated with a standardised >14-day definition for ‘definite’ nosocomial covid-19
- 3A: Excluding studies with a higher risk of bias (indicated by total quality score <5)
- 3B: Fulfilling all 5 core study quality domains (indicated by * within **Tables 2**–**4**).
- 4: Excluding studies with imputed data (i.e., 0.5 used in place of zero-count cells)
- 5: Studies utilising RT-PCR as the primary diagnostic method for SARS-CoV-2.

Additional data visualization was performed in R using the ggplot2 package.

### 2.6 Reporting Bias Assessment

Funnel plot and Egger’s test were used to assess for potential publication bias, supported by qualitative evaluation.

### 2.7 Certainty Assessment

The certainty of evidence was rated using the Grading of Recommendations, Assessment, Development and Evaluations (GRADE) approach (47) using the GRADEPro online tool, https://gradepro.org/.

## 3 Results

### 3.1 Study Selection and Characteristics

We screened a total of 1478 unique abstracts and reviewed 195 full texts to identify 67 studies describing hospital nosocomial COVID-19 outbreaks. Principal reasons for study exclusion are shown in **Figure 1**. A further 48 studies were excluded as they did not report mortality within both community and nosocomial-acquired COVID-19 patient groups. This left 21 studies for primary meta-analysis (9–13, 24–35, 37–40), summarised in **Table 1**, with both retrospective (n=14) and prospective (n=7) study designs including a range of medical and surgical patient populations. Together, these described 8251 hospitalised adults admitted between 1^st^ March 2020 and 13^th^ July 2020 across 7 countries, comprising 1513 (18.3%) probable or definite nosocomial COVID-19 and 6738 (81.7%) community-acquired cases. Overall mortality was 30.5% (2516/8251), with 572 deaths attributed to nosocomial COVID-19 (37.8% mortality rate) and 1944 (28.9% mortality rate) to community-acquired COVID-19. An additional study reporting the critical care admissions but without mortality by probable-nosocomial origin was identified, and is included **Table 1** (36).

### 3.2 Study Timing in Pandemic Course and Availability of Universal RT-PCR Testing

**Figure 2:**
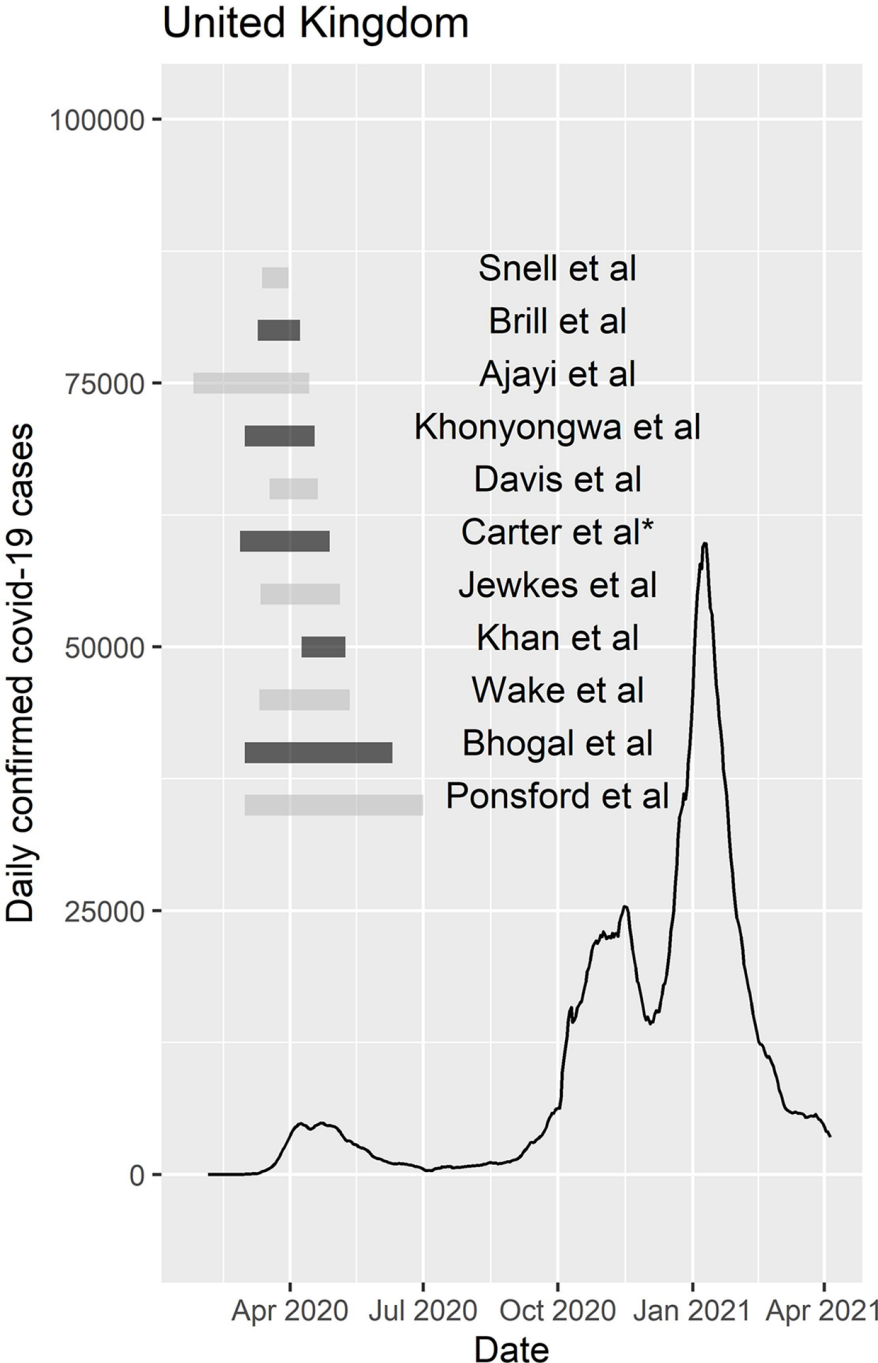
Timing of UK studies relative to national COVID-19 rates. Plot showing the timing of individual studies included within the primary meta-analysis reporting patients within the United Kingdom (UK), relative to national daily COVID-19 case diagnosis rates January 2020 and April 2021. * The study by Carter et al. is included here as 10/11 hospital sites were within the UK.

We explored the timing of patient identification within these reports relative to national COVID-19 diagnosis rates based on publicly available data within the UK (**Figure 2**), and wider countries (**Supplementary S2**). All included studies dealt with the initial wave of the pandemic. Consistent with the early timing of these reports, no studies reported the use of universal RT-PCR screening of patients in prior to or during admission from the outset of the study, outside of the setting nosocomial outbreaks.

### 3.3 Case Definitions

A positive reverse transcription polymerase chain reaction (RT-PCR) SARS-CoV-2 result was explicitly used as primary method of diagnosis in 17/21 studies included in the mortality meta-analysis (76%), supported by clinical-radiological features (12, 28, 40), or based upon laboratory-based diagnosis (potentially including serology) (27, 37). As shown in **Table 1**, a range of case definitions were employed to distinguish community-acquired and nosocomial COVID-19. A fixed interval between admission and diagnosis was employed in 14/21 (62%) ranging from >2 days (37) to >14 days (12), supplemented by additional patient-level clinical data (40) and viral whole genome sequencing (38). Seven studies primarily employed epidemiological nosocomial definitions, for instance a history of close contact with positive cases [n=3 (27, 31, 39)], or the absence of symptoms on admission with subsequent positive test [n=2 (10, 30)]. Two studies gave no explicit nosocomial case definition (24, 28). Four studies (19%) explicitly considered patients who had been recently discharged.

### 3.4 Risk of Bias in Studies

We screened study quality through self-identified use of reporting standards. Three (14%) reports referenced the STrengthening the Reporting of OBservational studies in Epidemiology (STROBE) statement (9, 12, 24). **Tables 2**–**4** show the formal risk of bias assessments. Overall, 17/21 (81.0%) achieved a total score of 5 or more. Using our more stringent assessment of study quality across all core domains (indicated by *) only 9/21 (43.0%) were identified, with 80% case series, 62.5% cohort, and 37.5% of prevalence rated at high risk of bias.

### 3.5 Meta-Analysis of Mortality in Patients With Nosocomial Relative to Community-Acquired COVID-19

**Figure 3:**
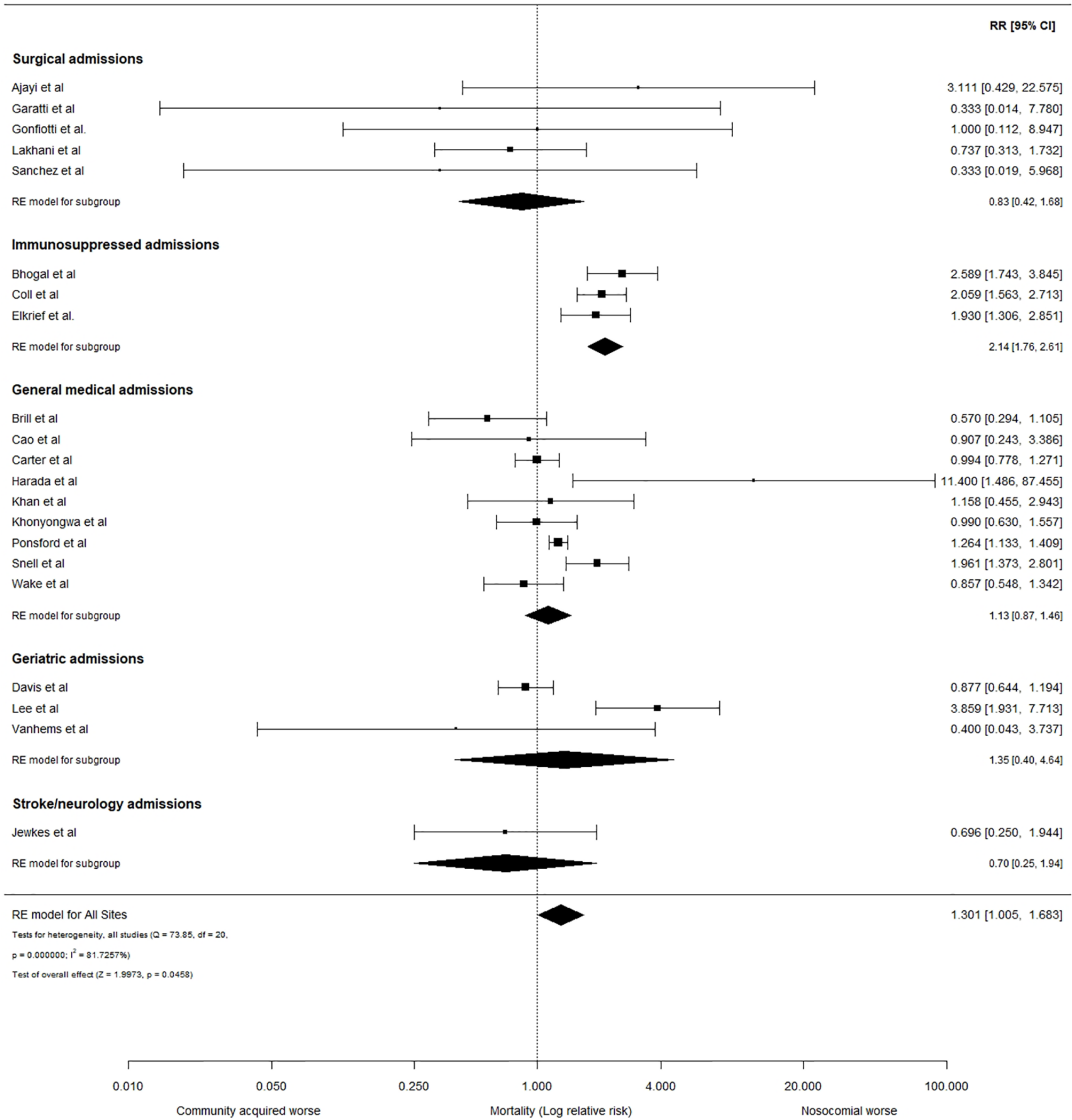
Relative risk of mortality in hospitalized adults with nosocomial and community-acquired COVID-19. Forest plot assessing the relative risk (RR) and 95% confidence interval (95% CI) of mortality in adults hospitalized with community-acquired and probable nosocomial COVID-19, according to the study definitions. The size of each box is proportional to the size of the individual hospital site (A-N), with the error bars representing the 95% CIs. The diamond represents the pooled average across studies, based on a random effects (RE) model. I^2^: heterogeneity variance, calculated using restricted effects maximum likelihood (REML).

Meta-analysis using a random effects model is shown in **Figure 3**. Across 21 studies, the risk of mortality was 1.301 (95% CI: 1.005 to 1.683) times greater in patients with probable or definite nosocomial infection, compared to those admitted with community-acquired COVID-19 (p=0.046). Substantial heterogeneity was evident between the included studies (Q= 73.8, p < 0.0001; I^2^ = 81.7%, 95% CI: 60.8 to 94.5%). We performed sub-grouping by patient cohort characteristics, including an immunosuppressed sub-group comprising 3 studies reporting outcomes from adult recipients of solid-organ or bone marrow transplants, or with a diagnosis of haematological or solid-organ cancers. These 1069 patients (152 nosocomial, 917 community-acquired) showed an elevated risk of death associated with nosocomial COVID-19, relative to those with community-acquired infection: RR= 2.14, 95% CI: 1.76 to 2.61 (p<0.0001). This effect appeared consistent across the 3 studies, but with considerable uncertainty associated with estimates of heterogeneity (Q= 1.24, p= 0.54; I^2^ = 0.00%, 95% CI: 0.00 to 96.6%). General medical (RR = 1.14, 95% CI: 0.87 to 1.46) and geriatric admissions (RR = 1.35, 95% CI: 0.40 to 4.64) were also suggestive of an increased risk of mortality with nosocomial COVID-19 but did not reach statistical significance (p=0.360 and 0.629, respectively).

### 3.6 Meta-Analysis of Critical Care Admission

**Figure 4:**
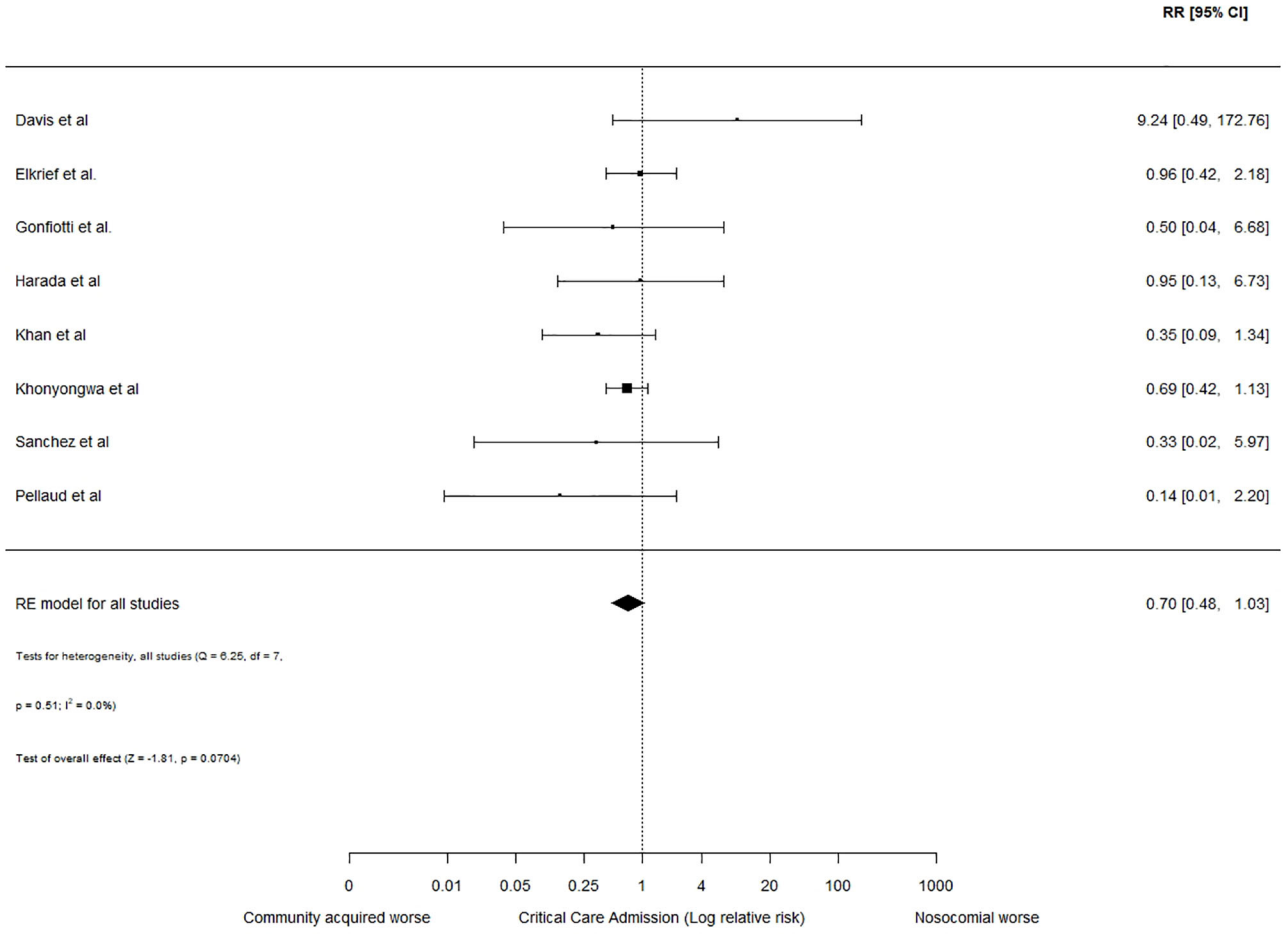
Relative risk of critical care admission in hospitalized adults with nosocomial and community-acquired COVID-19. Forest plot assessing the relative risk (RR) and 95% confidence interval (95% CI) of critical care admission in adults hospitalized with community-acquired and probable nosocomial COVID-19. The size of each box is proportional to the size of the individual hospital site (A-N), with the error bars representing the 95% CIs. The diamond represents the pooled average across studies, based on a random effects (RE) model. I^2^: heterogeneity variance, calculated using restricted effects maximum likelihood (REML).

Critical care admission rates were reported in 8 studies reporting nosocomial outbreaks (11, 13, 29, 31, 32, 34, 36, 37); with a crude rate of 27/252 (10.7%) in patients with nosocomial COVID-19 compared to 359/1396 (25.7%) in those hospitalised with community-acquired COVID-19. Meta-analysis is shown in **Figure 4**, with the pooled relative risk indicating this trend did not reach statistical significance (RR= 0.70, 95% CI: 0.48 to 1.03).

### 3.7 Sensitivity Analysis

To challenge the robustness of our findings, we examined the effect of varying the level of certainty of nosocomial case diagnosis, study quality, and use of imputed mortality data across 6 sensitivity analyses and assessed if individual studies conferred undue influence. These suggested that no individual study had undue influence on the results (**Supplementary S3**). Exclusion of studies across all sub-groups led to similar point estimates for the relative risk of mortality but did not reach statistical significance in 4 of 6 pre-specified analyses (p ≥ 0.05, see **Supplementary S3A**). Considering the immunosuppressed subgroup, the directionality and significance of our findings remained unchanged across 5 of 6 pre-specified sensitivity analyses (**Supplementary S3B**). Summary statistics for age were reported in 1287/1513 (85.1%) nosocomial cases (mean 77.3 years), and 4551/6738 (67.5%) community-acquired COVID-19 admissions (mean 70.1 years). Gender was available in 1309/1513 (86.5%) nosocomial cases (49.8% male) and 4846/6738 (71.9%) community-acquired COVID-19 admissions (56.5% male). Intra-study differences in age and gender, and lack of standardised summary data for factors such as co-morbidities, frailty, ethnicity, or deprivation precluded meta-regression analysis.

### 3.8 Reporting Biases

**Figure 5:**
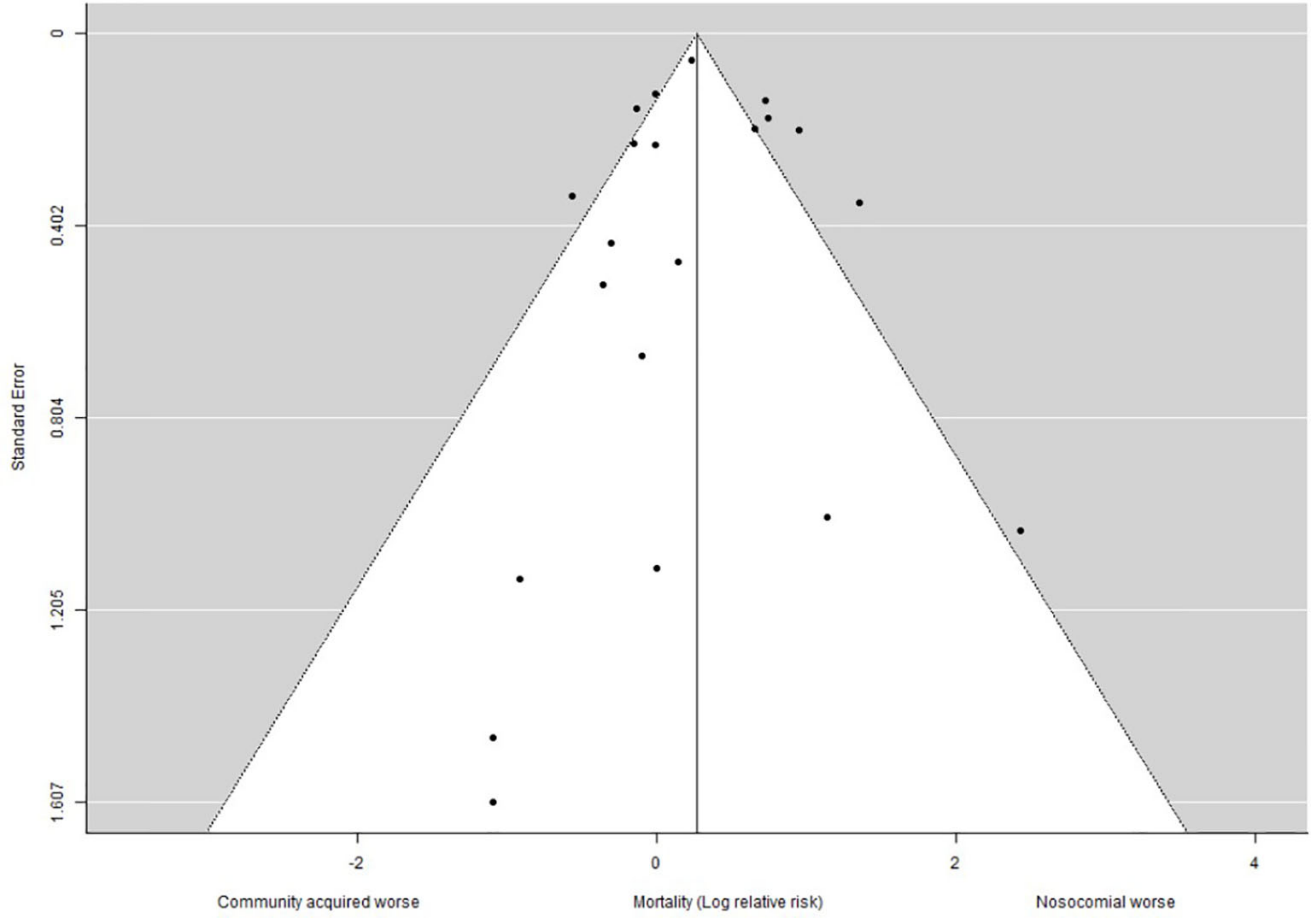
Funnel plot. Funnel plot with pseudo 95% confidence limits showing the distribution of relative risk of mortality across individual studies. Egger’s test, p = 0.51.

We assessed for publication bias by examining the cumulative evidence distribution for our primary outcome using a funnel plot (**Figure 5**). Egger’s test did not suggest funnel plot asymmetry (p=0.51). Given the potentially sensitive implications of nosocomial infection (48), we hypothesised selective reporting of mortality might exist between nations. We therefore compared the frequency and origin of reports identified at the full text eligibility review stage meeting our study definition of a nosocomial outbreak (n= 67), with those including mortality as an outcome within this patient group independent of community outcomes. Overall, 38 studies included mortality as an outcome (including 5 studies without observed nosocomial deaths), equating to a mortality reporting rate of 57%. **Table 5** shows variation in the rate of mortality reporting by country. Reports from the UK accounted for 21/67 (31%) of nosocomial reports and included mortality an outcome in 15/21 (71%). By contrast, reports from the United States contributed 7/67 (10%) of international reports describing nosocomial outbreaks, however none reported mortality as an outcome measure. This deviated significantly from the predicted international reporting rate (Fisher’s exact test, p = 0.0018). Together, this suggests publication bias may be present.

**Table 5:** Rates of mortality reporting in nosocomial COVID-19 outbreaks, by country of origin.

	Nosocomial outbreak reported		Nosocomial mortality reported as an outcome*	
Country	Total studies, n	Total of studies (%)	Included studies, n	Fraction of countries’ total reports
United Kingdom, UK	21	31%	15	71%
United States, US	7	10%	0	0%
China	6	9%	4	67%
Spain	5	7%	3	60%
France	3	4%	2	67%
Belgium	3	4%	0	0%
Italy	3	4%	2	67%
Switzerland	3	4%	1	33%
South Korea	2	3%	1	50%
Brazil	2	3%	2	100%
Japan	2	3%	2	100%
Vietnam	2	3%	0	0%
Germany	2	3%	1	50%
International	1	1%	1	100%
Poland	1	1%	1	100%
Denmark	1	1%	0	0%
India	1	1%	1	100%
Canada	1	1%	1	100%
Ireland	1	1%	1	100%
**Total**	**67**	**-**	**38**	57%

### 3.9 Certainty of Evidence

We assessed the quality of evidence supporting the statement: “In the general adult population, nosocomial COVID-19 is associated with a greater risk of inpatient mortality compared to individuals hospitalised with community-acquired COVID-19” as very low; and low/moderate in relation to “In an immunosuppressed adult population, nosocomial COVID-19 is associated with a greater risk of inpatient mortality compared to individuals hospitalised with community-acquired COVID-19”. Full GRADE assessment is shown in **Table 6**.

**Table 6:** Grading of Recommendations, Assessment, Development and Evaluations (GRADE) assessment.

Statement	Number of studies and patients	Risk of bias	Indirectness	Inconsistency	Imprecision	Other considerations	Effect size	Overall quality of evidence
“In the general adult population, nosocomial COVID-19 is associated with a greater risk of inpatient mortality compared to individuals hospitalised with community-acquired COVID-19”	21 studies, 8251 patients. Probable nosocomial: 1513 Probable community: 6738	Serious - Very serious	Not serious	Very serious	Not serious	Publication bias suspected ^2^	RR 1.301 95% CI: 1.005 to 1.683	Low/very low
“In an immunosuppressed adult population, nosocomial COVID-19 is associated with a greater risk of inpatient mortality compared to individuals hospitalised with community-acquired COVID-19”	3 studies, 1069 patients. Probablenosocomial: 152 Probable community: 917	Serious*	Not serious	Not serious	Serious^1^	Publication bias suspected ^2^ Strong association ^3^	RR 2.14 95% CI: 1.76 to 2.61	Low/Moderate

Created using GRADEPro online tool, https://gradepro.org/. * All studies scored moderate/high in formal assessment; however, follow-up duration was limited; ^1^ Significant uncertainty associated with heterogeneity assessment: I^2^ = 0.00%, 95% CI: 0.00 to 96.6%, downgrade by 1 level; ^2^ Mortality reporting bias suspected by country, downgrade by 1 level; ^3^ RR > 2.0 with consistent effect from ≥2 studies, upgrade by 1 level.

## 4 Discussion

In this systematic review and meta-analysis addressing the burden of nosocomial COVID-19, we show the case fatality rate for nosocomial COVID-19 appears greater than community-acquired COVID-19, with a relative risk of 1.301 (95% CI: 1.005 to 1.683). Strikingly, we found that patients with malignancy (11, 25) or transplant recipients (28) had approximately double the risk of dying after acquiring COVID-19 in hospital, compared to those hospitalised with community-acquired infection. This equates to a crude absolute inpatient mortality rate of 50.7% *vs.* 23.8% respectively, with a consistent effect across studies which proved robust to sensitivity analyses assessing multiple assumptions around the certainty of nosocomial COVID-19 diagnosis and study quality.

The convergence of widely recognized risk factors for adverse outcomes in community-acquired COVID-19 in hospitalised patient groups, such as advanced age and frailty, are likely to contribute to the exaggerated mortality burden observed with nosocomial COVID-19. A range of potential mechanisms are likely to link individuals with cancer or recipients of transplants with mortality risk from nosocomial COVID-19, including both immunosuppression linked to the underlying condition and/or treatments and exposure due to health care requirements necessitating admission to the acute hospital environment. This is convergent with the heightened risk of mortality from COVID-19 reported for individuals with inherited and acquired forms of immunodeficiency (16), and the wider susceptibility of patients with haematological malignancy across a spectrum of healthcare-associated infections (49). Individual studies suggested a relationship between mortality rates and degree of immunosuppression, with the greatest mortality rate observed in patients with haematological malignancies who had recently received chemotherapy (25). This is consistent with results from patients enrolled within the UK Coronavirus Cancer Monitoring Project, which included 227 patients with haematological malignancies diagnosed with COVID-19 (50). In this setting, recent chemotherapy approximately doubled the odds of dying during COVID-19-associated hospital admission (odds ratio: 2.09; 95% CI 1.09 to 4.08) after adjusting for age and gender; however, this study did not account for nosocomial infection (50). Conflicting outcomes in the haematopoietic stem cell transplantation (HSCT) population following COVID-19 are reported (51, 52). The largest multicentre study to date followed 318 patients, suggesting 15% of allogeneic and 13% of autologous HSCT recipients developed severe COVID-19; overall survival in both HSCT-groups was approximately 70% at 30-days following COVID-19 diagnosis (52).

Our study has several strengths. We systematically screened both the peer-reviewed and pre-print literature, leveraging the enhanced availability of full-texts by many publishers, to summarise the outcomes of 8251 adults hospitalised with COVID-19 during the first wave of the pandemic across 8 countries. This work establishes a relevant baseline for subsequent and future waves of the COVID-19 pandemic, and to our knowledge, represents the first meta-analysis of nosocomial COVID-19 mortality rates published to date. Zhou et al. reported a rapid review and meta-analysis of nosocomial infections due to a range of viral pandemic threats, but included only 3 studies with SARS-CoV-2 and did not consider mortality as an outcome (7). To support the generalisability of our findings, we included studies with implicit and explicit definitions of nosocomial COVID-19. Accordingly, we catalogued a wide spectrum of case definitions, including combined epidemiological and genomic viral sequencing (38). We controlled for this variation in case definitions within our sensitivity analyses, for instance using outcomes meeting consensus international criteria for definite nosocomial infection wherever available. Although our funnel plot did not indicate publication bias amongst studies reporting mortality, our sequential literature review process suggests variation in the frequency of mortality reporting associated with studies describing nosocomial COVID-19 outbreaks. In particular, we identified no studies reporting mortality associated with nosocomial COVID-19 infection outbreaks originating from the United States, despite the high rate of COVID-19 cases and mortality in this country to date (53). Of the 7 studies we identified reporting nosocomial COVID-19 at the full text review stage, four dealt only with incidence (54–57), whilst three reported mortality but without reference to probable origin (58–60). Whilst we cannot exclude the risk of reporting bias, given the sensitive nature of this topic (48), this observation highlights successful infection control practices. Reporting on experience from a large US academic medical centre, Rhee et al. found that despite a high burden of COVID-19, only two patients likely acquired COVID-19 during their admission (54). Generalising these practices may constitute a challenge across global health care settings acutely, for instance shortages of negative pressure isolation rooms were reported during the first wave in UK hospitals (34), but remain relevant as part of a longer-term “rebuild better” strategy.

Our study also has limitations, including its focus on hospitalised patients during the first wave of the pandemic. This is likely to introduce both selection and reporting bias, as during this period limited capacity meant RT-PCR testing was initially restricted to symptomatic individuals in the community (33, 40). Estimates of age-stratified infection fatality rates in the adult UK general population during the first wave ranged from 0.03% (20-29 years) to 7.8% (over 80 years) (61), far lower than the inpatient comparator mortality rate used in our analysis. By contrast, individuals admitted during nosocomial outbreaks were more likely to be subject to screening, resulting in sampling of individuals across the true spectrum of disease severities (29, 34), including earlier in their disease course. Our risk of bias assessment therefore focused on study inclusion and adequate follow-up as essential domains, to account for unequal disease progression at study entry between groups. It is also important to appreciate that as studies typically reported all-cause mortality - and information on age, frailty, and co-morbidities were not available at the individual patient level - the causal contribution of nosocomial COVID-19 exposure remains to be determined. Examination of linked primary care and mortality data within the United Kingdom (62, 63) suggests that COVID-19 amplifies the risk of death by a factor associated with the levels of circulating virus and an individuals’ underlying diagnoses (62). Shah et al. describe how active SARS-CoV-2 infection often led to decisions to forgo anticancer treatment in hospitalised patients with haematological malignancies (51). Together this illustrates the intricate relationship by which nosocomial circulation of SARS-CoV-2 and comorbidities together contribute to increase the risk of mortality. Surveillance schemes based on standardised case definitions, assessment of co-morbidities, and estimation of excess mortality are required to better explore this relationship.

In conclusion, we systematically gathered data from the international literature to describe the risk of inpatient mortality associated with nosocomial and community COVID-19. In particular, we strengthen observational evidence indicating individuals with malignancy or transplant recipients are at markedly elevated risk of death when infected by SARS-CoV-2 in hospital, compared to the community. This maybe underestimated due to consideration of only hospitalised individuals. With the continued occurrence of new viral variants with enhanced transmissibility and severity, SARS-CoV-2 appears likely to become an endemic virus. Our findings are likely of ongoing significance despite vaccination, given confirmation of an impaired SARS-CoV-2 vaccine response in multiple patient groups (64–67). Meanwhile, vaccination does not provide sterilising immunity in the immunocompetent, with vaccinated healthcare workers demonstrated to shed SARS-CoV-2 virus (68), creating conditions for continued nosocomial transmission. Together, these findings inform policy makers by strongly advocating continued public health surveillance, stringent infection control measures (54), and access to individualised clinical interventions such as pre- or post-exposure immuno-prophylaxis with monoclonal antibodies targeting the anti-SARS-CoV-2 spike protein (69, 70) to combat the threat of nosocomial COVID-19.

## Data Availability Statement

The original contributions presented in the study are included in the article/**Supplementary Material**. Further inquiries can be directed to the corresponding author.

## Author Contributions

MP conceived the project and drafted the protocol with TW and SS, with supervision from SB, SJ, IH, and DF. MP, SS, TW, KO, CD, and DS screened abstracts and performed the full text review. MP, TW and SS performed the data quality assessment. TW and MP analysed the data. MP prepared the first draft of the manuscript. All authors contributed to the article and approved the submitted version.

## Funding

This work was partly funded by UKRI/NIHR through the UK Coronavirus Immunology Consortium (UK-CIC). MP is supported by the Welsh Clinical Academic Training (WCAT) programme and a Career Development Award from the Association of Clinical Pathologists and is a participant in the NIH Graduate Partnership Program. IH is a Wellcome Trust Senior Research Fellow in Basic Biomedical Sciences. The funding sources did not have any role in designing the study, performing analysis, or communicating findings. TW is supported by an NIHR Clinical Lectureship. This research was funded in part by the Wellcome Trust. For the purpose of open access, the authors have applied a CC BY public copyright licence to any Author Accepted Manuscript version arising from this submission.

## Conflict of Interest

The authors declare that the research was conducted in the absence of any commercial or financial relationships that could be construed as a potential conflict of interest.

## Publisher’s Note

All claims expressed in this article are solely those of the authors and do not necessarily represent those of their affiliated organizations, or those of the publisher, the editors and the reviewers. Any product that may be evaluated in this article, or claim that may be made by its manufacturer, is not guaranteed or endorsed by the publisher.
