# Supplementary material for: A Systematic Review and Meta-Analysis of Inpatient Mortality Associated With Nosocomial and Community COVID-19 Exposes the Vulnerability of Immunosuppressed Adults
Source: Front Immunol. 2021 Oct 6;12:744696. doi: 10.3389/fimmu.2021.744696 (PMC8526940; doi:10.3389/fimmu.2021.744696)
Supplement: Supplementary file 1 [file DataSheet_1.pdf]

Supplementary online material

Contents

Supplementary S1: Extended Methods 2

Supplementary S2: Timing of non-UK studies included within primary meta-analyses relative to national COVID-19 rates 3

Supplementary S3: Sensitivity Analyses 4

Supplementary S4: Risk of bias assessment tools 5

A: Newcastle-Ottawa Score (cohort studies) 5

B: Joanna Briggs Institute (JBI) tool (cross-sectional/prevalence studies) 6

C: Joanna Briggs Institute (JBI) tool (case series) 6

Supplementary S1: Extended Methods

Individual study variance and heterogeneity variance were combined to calculate individual study weight: $w_i = 1/(\tau^2 + \sigma^2)$. Studies which reported no observed deaths in both community acquired or nosocomial COVID-19 groups were excluded from the analysis. When only one group reported no deaths, a figure of 0.5 deaths was used for the purpose of analysis. Assumption of normality for meta-analysis models was assessed using Q-Q plots. To establish whether an individual study had undue influence on the meta-analysis model, the 'influence' function in the R metafor package was used. Studies were judged influential if one of the following was true:

- The absolute DFFITS value is larger than $3\sqrt{p/(k-p)}$, where p is the number of model coefficients and k the number of studies.
- The lower tail area of a chi-square distribution with p degrees of freedom cut off by the Cook's distance is larger than 50%.
- The hat value is larger than 3(p/k).
- Any DFBETAS value is larger than 1.

We pre-specified the following sensitivity analysis:

- 1: Studies providing an explicit definition of nosocomial acquisition
- 2: Studies providing outcomes associated with a standardised >14 day definition for 'definite' nosocomial covid-19 (excluding probable cases).
- 3A: Excluding studies with a higher risk of bias, defined as studies with a score of 4 or less.
- 3B: Fulfilling all 5 core study quality domains (as indicated by * within tables 2-4 or Supplementary S4).
- 4: Excluding studies with imputed data (i.e. 0.5 used in place of zero-count cells)

Supplementary S2: Timing of non-UK studies included within primary meta-analyses relative to national COVID-19 rates

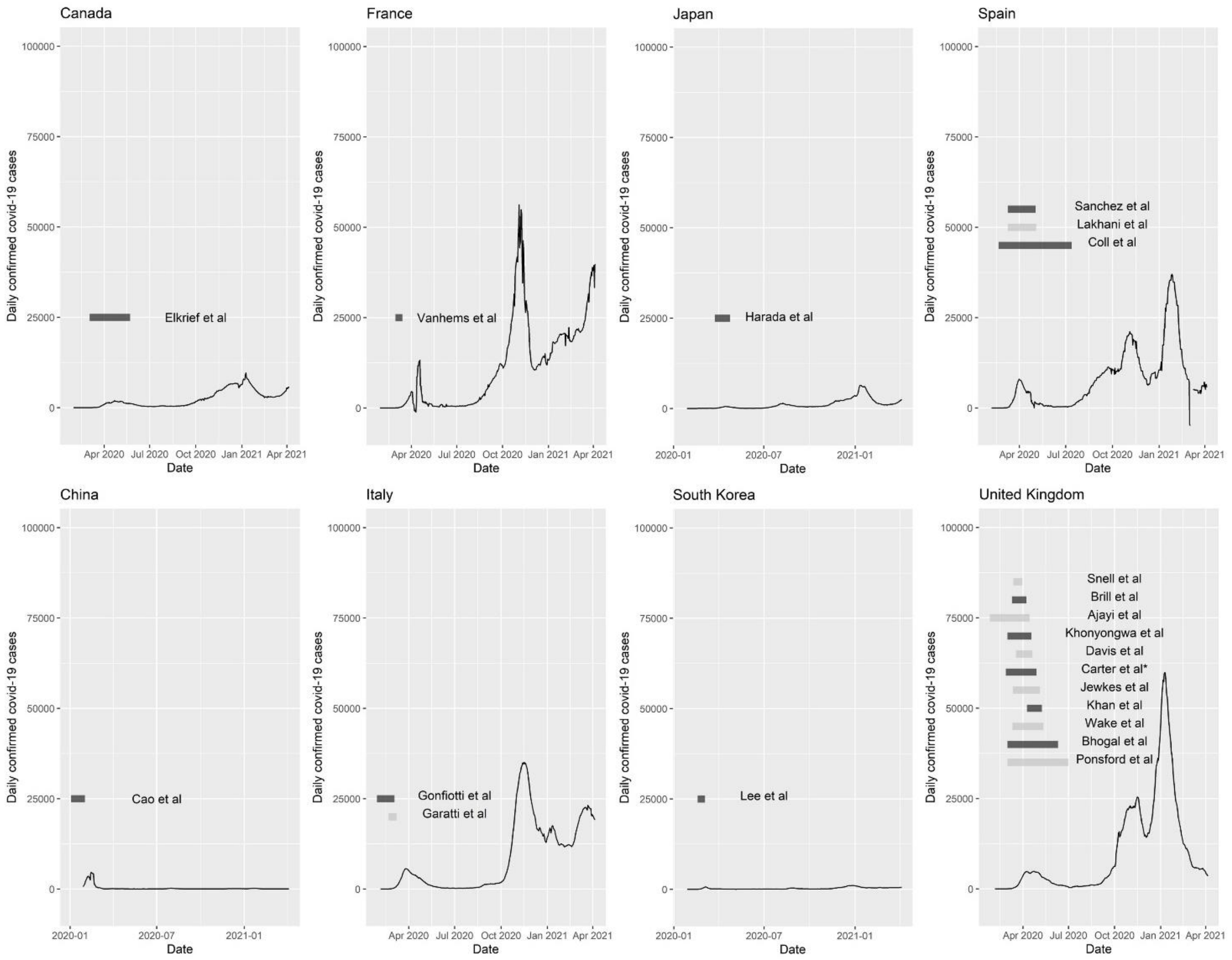

Supplementary S3: Sensitivity Analyses

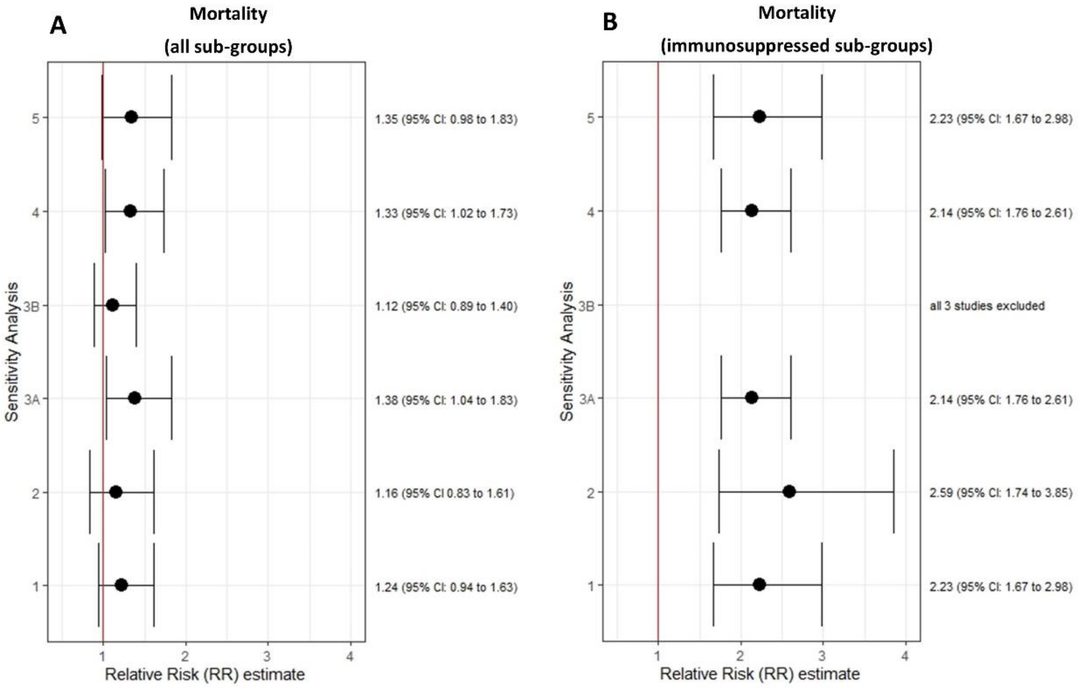

Forest plots showing the relative risk (RR, log estimate) and 95% confidence interval (95% CI) estimates for the relative risk of mortality in adults hospitalised with community-acquired and probable nosocomial COVID-19 applying pre-defined sensitivity analyses across **A:** subgroups or to **B**: immunosuppressed sub-group only. **1**: Studies providing an explicit definition of nosocomial acquisition; **2**: Studies providing outcomes associated with a standardized >14 day definition for 'definite' nosocomial covid-19; **3A**: Excluding studies with a higher risk of bias (indicated by total quality score <5); **3B**: Fulfilling all 5 core study quality domains (indicated by * within tables 2-4, see main article); **4**: Excluding studies with imputed data (i.e. 0.5 used in place of zero-count cells). **5**: Considering only studies utilising RT-PCR as the primary diagnostic method for SARS-CoV-2.

There was no significant difference in the overall mortality analysis when studies without an explicit definition of nosocomial acquisition were excluded (n=19, p=0.78), however, removing these studies changed the overall relative risk for the difference between mortality from nosocomial and community acquired COVID-19 such that it no longer met the pre-defined 5% significance level (RR = 1.24, 95% CI 0.94 to 1.63, p= 0.13). There was no significant difference in the analysis when only studies providing outcomes based on standardised >14 day definition for 'definite' nosocomial covid-19 were included (n = 8, p= 0.54), however the difference between mortality from nosocomial and community acquired COVID-19 no longer met the pre-defined 5% significance level (RR = 1.15, 95% CI 0.83 to 1.58, p= 0.40). There was no significant change in mortality outcome when studies with a with "raw" risk of bias score of 5 or less were excluded (n=4, RR = 1.39 vs 1.31, p=0.76). There was no significant difference in the analysis when studies with high risk of bias were excluded (defined as studies scoring across all key 5 domains, indicated by * in Tables 2-4), n=12, p=0.42, however the difference between mortality from nosocomial and community acquired COVID-19 no longer met the pre-defined 5% significance level (RR = 1.13, 95% CI 0.88 to 1.45, p = 0.34). Excluding studies where data was imputed (n=3) had no significant effect on the results (RR 1.34 vs 1.31 p=0.91).

Supplementary S4: Risk of bias assessment tools

A: Newcastle-Ottawa Score (cohort studies)

*1) Representativeness of the exposed cohort [“nosocomial covid-19”]
a) truly representative of the average nosocomial covid-19 case in the hospital (or wider patient group), 1
b) somewhat representative of the average nosocomial covid-19 case in the hospital (or wider patient group), 0
c) selected group of users e.g. single ward or department; possible inclusion of children, nurses, volunteers, 0
d) no description of the derivation of the cohort, 0
*2) Selection of the comparator cohort [“community” covid-19 infection]
a) drawn from the same population (e.g. ward, hospital, region) as the exposed [nosocomial] cohort,1
b) drawn from a different source (e.g. national dataset),0
c) no description of the derivation of the non-exposed cohort, 0
*3) Ascertainment of nosocomial covid-19 infection
a) secure record (e.g. medical records), 1
b) structured interview, 1
c) written self report, 0
d) no description,0
4) Demonstration that outcome of interest was not present at start of study (SARS-CoV-2 infection prior to admission)
a) yes (universal screening present on admission), 1
b) no, 0
**Comparability:**
5) Comparability of cohorts on the basis of the design or analysis (for end-point of mortality)
a) study controls for age, 1
b) study controls for any additional factor (e.g. frailty, or comorbidities differences between patient groups, e.g. multiple regression), 1
c) study controls for multiple important factors, 2
d) no attempt to control for relevant factors, 0
**Outcome:**
6) Assessment of outcome
a) independent blind assessment, 1
b) record linkage, 1
c) self report, 1
d) no description, 1
*7) Was follow-up long enough for outcomes to occur (28 days or until discharge of diagnosis)
a) Yes*, 1
b) no (or not reported), 0
*8) Adequacy of follow up of cohorts
a) complete follow up - all subjects accounted for, 1

b) subjects lost to follow up unlikely to introduce bias, 1
c) follow up rate < 80% and no description of those lost, 0

B: Joanna Briggs Institute (JBI) tool (cross-sectional/prevalence studies)

Domain 1*	Were criteria for inclusion in the sample clearly defined?
Domain 2*	Were the study subjects and setting described in detail?
Domain 3*	Was the exposure (case definition nosocomial/community COVID-19) measured in a valid and reliable way?
Domain 4*	Were objective, standard criteria used for measurement of the condition? (use of secure medical records or linked datasets, e.g. RT-PCR testing records)
Domain 5	Were confounding factors identified? (e.g. Health care workers vs patients, age, frailty, treatments)
Domain 6	Were strategies to deal with confounding factors stated?
Domain 7*	Were the outcomes measured in a valid and reliable way? (mortality reporting based on follow-up 28 days since onset, or until death or discharge)
Domain 8	Was appropriate statistical analysis used? (e.g. use of multivariate analysis, at minimum: age-stratified mortality by nosocomial vs community COVID-19 origin)

C: Joanna Briggs Institute (JBI) tool (case series)

Domain 1*	Were there clear criteria for inclusion in the cases series?
Domain 2*	Was the condition measured in a standard, reliable way for all participants (use of a standardised case definitions for community and nosocomial)
Domain 3	Were valid methods used for identification of the condition for all participants (e.g. RT-PCR diagnosis)
Domain 4*	Did the case series have consecutive inclusion of participants
Domain 5	Did the case series have complete inclusion of participants
Domain 6	Was there clear reporting of the demographics of the participants in the study
Domain 7	Was there clear reporting of the clinical information of the participants
Domain 8*	Were the outcomes or follow up results of cases clearly reported (target: 28 days since onset, or until death or discharge)
Domain 9*	Was there clear reporting of the presenting sites(s)/clinic(s) demographic information
Domain 10	Was the statistical analysis appropriate
